# Costs of Raising Grandchildren on Grandmother-Adult Child Relations in Black and White Families

**DOI:** 10.1093/geroni/igab046.1524

**Published:** 2021-12-17

**Authors:** Yifei Hou, J Jill Suitor, Megan Gilligan, Destiny Ogle, Catherine Stepniak, Yufu Jiang

**Affiliations:** 1 Purdue University, West Lafayette, Indiana, United States; 2 Purdue University, Purdue University, Indiana, United States; 3 Iowa State University, Iowa State University, Iowa, United States

## Abstract

The cost of raising grandchildren on grandmothers’ mental and physical health has been well-documented; however, little is known about whether raising grandchildren also has a cost on grandmothers’ relationships with the adult children whose children the grandmothers have raised. Drawing from theories of exchange and affect, stress process model, and racial differences in intergenerational solidarity, we tested how raising grandchildren affects grandmother-adult child relations. Further, we explored the extent to which these patterns differed by race. To address this question, we used mixed-methods data collected from 553 older mothers regarding their relationships with their 2,016 adult children; approximately 10% of the mothers had raised one or more of their grandchildren “as their own.” Data were provided by the Within-Family Differences Study-I. Multilevel analyses showed that raising grandchildren was associated with greater closeness in grandmother-adult child relationship in Black families; however, in White families, raising grandchildren was associated with greater conflict in the grandmother-adult child relationship. Further, the differences by race in the effects of raising grandchildren on closeness and conflict were statistically significant. Qualitative analyses revealed that race differences in the association between raising grandchildren and relationship quality could be explained by mothers’ reports of greater family solidarity in Black than White families. Our findings highlight the ways in which race and family solidarity interact to produce differences in the impact of raising grandchildren on Black and White mothers’ assessment of the quality of their relationships with their adult children, consistent with broader patterns of racial differences in intergenerational cohesion.

